# Editorial: Genome editing applications of CRISPR/Cas9 in metabolic diseases, hormonal system and cancer research

**DOI:** 10.3389/fendo.2023.1256966

**Published:** 2023-08-07

**Authors:** Pengpeng Liu, Lei Huang, Chun-Qing Song

**Affiliations:** ^1^ Molecular, Cell and Cancer Biology (MCCB), 1University of Massachusetts Medical School, Worcester, MA, United States; ^2^ Institute of Basic Medical Sciences, Westlake Institute for Advanced Study, Hangzhou, Zhejiang, China

**Keywords:** CRISPR/Cas9, genome editing, metabolic diseases, hormonal system disease, cancer research

Genome editing is an advanced technology that enables scientists to make precise alterations to the DNA of living cells, holding great potential for transforming disease treatment, crop development, and even the creation of novel life forms. Among the most promising genome editing techniques is the CRISPR/Cas9 system, which has been modified to be programmable and target specific genes in any organism (1–3).

Metabolic diseases, hormonal system-related disorders, and cancer are complex conditions that can lead to various health issues, including obesity, diabetes, heart disease, and stroke. These diseases can result from genetic mutations, environmental factors, or a combination of both. CRISPR/Cas9 shows promise in treating metabolic diseases through multiple approaches (4–6).

Meng et al. summarized the potential applications of CRISPR/Cas9 in the following areas: (1) Correcting genetic mutations responsible for metabolic diseases. For instance, CRISPR/Cas9 could be employed to rectify mutations in the gene responsible for the insulin receptor, thereby aiding in the treatment of type 2 diabetes. (2) Introducing new genes that enhance the body’s ability to metabolize food more efficiently. For instance, CRISPR/Cas9 could be used to insert genes encoding enzymes that improve carbohydrate or fat breakdown. (3) Disabling genes involved in the development of metabolic diseases. For instance, CRISPR/Cas9 could be utilized to deactivate genes that produce proteins promoting inflammation or insulin resistance. The authors also discussed potential applications of CRISPR/Cas9 in the treatment of hormonal disorders.

Wei et al. reviewed the potential application of CRISPR/Cas9 in various diseases, including cancer. Specifically, they highlighted key findings regarding CRISPR/Cas9 and HPV-driven cancer: (1) CRISPR/Cas9 can target and eliminate the HPV genes responsible for cancer. (2) CRISPR/Cas9 has demonstrated effectiveness in treating HPV-driven cancer in mice. (3) Clinical trials are currently underway to evaluate the safety and efficacy of CRISPR/Cas9 in treating HPV-driven cancer in humans. While research on CRISPR/Cas9 and HPV-driven cancer is still in early stages, the potential for this technology to cure cancer is promising.

Lin et al. reported the discovery of CS271011, a liver-targeted thyroid hormone receptor-β agonist. CS271011 has been found to reduce liver steatosis (accumulation of fat in the liver) and improve hepatic lipid metabolism in mice with diet-induced obesity. This paper presents promising evidence that CS271011 could potentially treat lipid metabolism disorders such as non-alcoholic fatty liver disease (NAFLD). However, further research is necessary to confirm these findings and evaluate the safety and efficacy of CS271011 in humans.

Ma et al. investigated the effects of glycated transferrin (GTF) on HK-2 cells, which are liver cells. GTF is a modified form of transferrin that has undergone glucose-related modifications. The study found that GTF induced apoptosis (programmed cell death) in HK-2 cells and reduced the expression of genes associated with cell growth and survival. The authors concluded that GTF could be a contributing factor to the development of diabetic complications, such as liver damage. However, more research is needed to validate these findings and understand the long-term effects of GTF.

As technology continues to advance, it is likely that CRISPR/Cas9 will be utilized in treating a wider range of diseases in the future. This technology holds the potential to significantly impact human health and stands as one of the most promising developments in biomedical research (7, 8). However, there are still challenges that must be addressed before CRISPR/Cas9 can be used in clinical trials. These challenges include: (1) Ensuring the specificity of CRISPR/Cas9, as it can sometimes target unintended genes, leading to undesirable side effects. (2) Enhancing the efficiency of CRISPR/Cas9, as it may not always achieve the desired DNA modifications. (3) Assessing the long-term safety of CRISPR/Cas9, as its potential for causing health problems remains unknown. Thus, additional research is necessary to overcome these challenges and fully exploit the potential of CRISPR/Cas9 in clinical trials for metabolic diseases, hormonal system-related disorders, and cancer (4, 9, 10).

## Author contributions

PL: Writing – original draft, Writing – review & editing. LH: Writing – review & editing. CS: Writing – review & editing.
